# Combination of Polydopamine and Plasma Oxidation to Increase Tissue Integration of Polyurethane‐Silicone Copolymers for Cardiovascular Implants

**DOI:** 10.1002/adhm.202500577

**Published:** 2025-06-25

**Authors:** Sophie Armstrong, Jennifer M. Dyson, Lingxiao Zeng, Saeedreza Zeibi Shirejini, John S. Forsythe, Shaun D. Gregory

**Affiliations:** ^1^ CardioRespiratory Engineering and Technology Laboratory (CREATElab) Department of Mechanical and Aerospace Engineering Monash University Melbourne VIC 3800 Australia; ^2^ Department of Materials Science and Engineering Monash University Melbourne VIC 3800 Australia; ^3^ Cancer Program Department of Biochemistry and Molecular Biology Biomedicine Discovery Institute Monash University Melbourne VIC 3800 Australia; ^4^ Monash Centre for Additive Manufacturing Monash University Notting Hill VIC 3168 Australia; ^5^ Australian Centre for Blood Diseases School of Translational Medicine (STM) Monash University Melbourne Victoria 3004 Australia; ^6^ Centre for Biomedical Technologies and School of Mechanical Medical and Process Engineering Queensland University of Technology Brisbane QLD 4000 Australia

**Keywords:** elasteon, plasma oxidation, polydopamine deposition, polyurethane co‐polymers

## Abstract

Polyurethane (PU)‐silicone co‐polymers are increasingly favored in medical applications due to their excellent biostability and durability; however, their intrinsic hydrophobicity limits tissue integration. Polydopamine (PDA) deposition is a widely accepted method for increasing biomaterial surface hydrophilicity, though concentrations and methods vary across published literature. This study investigates the synergistic effects of PDA deposition and plasma oxidation on FDA‐approved Elast‐Eon E2A (E2A) to enhance cell attachment and wound healing. E2A substrates are treated with a range of plasma oxidation periods and PDA concentrations (0–5 min, 0‐0.5 w v−1% respectively). The combination of 0.05 w v−1% PDA and 1‐minute oxygen plasma results in the most significant reduction in water contact angle (92to 19°), increase in fibroblast adhesion (33.0–53.2 cells mm^−2^) and cell diameter, with an overall increase in intra‐ and extracellular collagen I and fibronectin. X‐ray photoelectron spectroscopy (XPS) reveals significant surface chemical changes, while surface roughness remains unchanged. Whole blood adhesion tests show no change in platelet adhesion or volume. These parameters may offer an improved approach for modifying PU‐copolymers to enhance cell interactions for use in current and future medical implants, including a suite of cardiovascular technologies that require both material ductility and rapid tissue integration.

## Graphical abstract

Plasma oxidation following polydopamine deposition can significantly reduce the surface hydrophobicity of polyurethane‐siloxanes without impacting surface roughness, thereby increasing fibroblast viability, adhesion, and extracellular matrix production and secretion. This method is suggested to be an enhanced approach to increasing the tissue integration of medical polyurethane copolymers, particularly those used in cardiovascular implants.

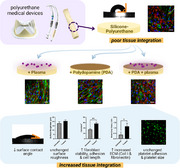

## 1 Introduction

Biomaterials that are implanted to replace dysfunctional or missing tissue, or as part of a medical device, provide a substrate for proliferation and act as a mechanical support for regeneration.^[^ ^1^ ^]^ To prevent mechanical failure of the construct or necrosis of surrounding tissue, the construct should closely match the mechanical properties of native tissues. Contractile tissues (such as those found in the cardiovascular system) exhibit anisotropic, viscoelastic behavior, and hence require highly flexible and tough implants.

Traditionally, flexible biomaterials have been made of polysiloxanes, namely polydimethylsiloxane (PDMS), and polyurethanes (PU) as a result of their excellent biocompatibility, bio‐durability, sterilisability, processability, and tunable mechanical properties.^[^ ^2^ ^]^ PUs are block copolymers and the limited biostability of the material can be improved by replacing some of the polyethers with a siloxane segment, as PDMS is more resistant to oxidative degradation.^[^ ^3^ ^]^ For example, artificial heart valves made from Elast‐Eon demonstrate a durability of 500 million cycles.^[^ ^4^ ^]^

Elast‐Eon E2A (E2A) is an FDA‐approved silicone‐polyurethane (PU) co‐polymer with greater than 450% elongation at break and 70–90 kN m^−1^ tear strength which shows promise for use in cardiovascular applications.^[^ ^5^ ^]^ However, a major limitation in instances where rapid tissue integration of polyurethane co‐polymers is desirable – such as with transcutaneous drivelines in order to prevent infection – is the low‐energy hydrophobic surface impeding cell attachment. Furthermore, polyurethanes are of particular interest in blood‐contacting devices. In this instance their low surface energy can reduce protein adsorption and platelet adhesion. However, this hydrophobic nature can inhibit cell adhesion, which is critical for the long‐term performance and integration of blood‐contacting devices. Therefore, achieving an optimal balance between surface hydrophilicity and hydrophobicity is essential to promote both hemocompatibility and endothelialization.

Polydopamine (PDA) deposition is a common method to modify the surface energy of biomaterials, with high efficiency and repeatability at a relatively low cost.^[^ ^6^, ^7^ ^]^ PDA, a biomimetic, self‐adherent polymer, undergoes oxidative polymerization in alkaline conditions with strong adsorption to a wide variety of surfaces through covalent bonding and intermolecular forces.^[^ ^8^ ^]^ Surface oxygen plasma treatment is another popular modification technique, either to increase bonding with a bioactive molecule or to increase interactions with cells directly.^[^ ^9^, ^10^ ^]^ Furthermore, plasma oxidation is a well‐known method of reducing the thrombosis risk of cardiovascular biomaterials.^[^ ^11^ ^]^

A review of PDA processing parameters for tissue engineering applications suggests that 2 mg mL^−1^ (0.2 w v^−1^%) PDA is required to achieve a complete layer, polymerized in pH 8.5 Tris‐HCl for 24 h at ambient temperature, although cell proliferation over two weeks was independent of coating time.^[^ ^5^, ^8^ ^]^ Ghorbani et al. reported a significant increase in PDA deposition after 90‐s oxygen plasma treatment which in turn increased cell viability and alkaline phosphatase activity – a marker of osteodifferentiation – at 48 and 96 h.^[^ ^12^ ^]^

Therefore, this study aimed to investigate E2A as a biomaterial candidate when treated with a combination of PDA deposition and plasma treatment as a versatile surface engineering protocol for enhanced tissue integration. A range of PDA concentrations were examined, both pre‐and post‐plasma treatment, with 24 h coating time used in all experiments. Surfaces were characterized using water contact angle, X‐ray photoelectron spectroscopy (XPS), and optical profilometry to reveal the change in surface chemistry, morphology, and energy. Finally, the impact of surface engineering on human fibroblast attachment, morphology, and ECM production is explored, to better understand how PU co‐polymers may be modified for use in current and future medical implants with tissue‐like mechanical properties. These experiments may provide an improved approach to a cost‐effective and simple high‐throughput method of increasing the biocompatibility of polyurethane co‐polymers.

## 2 Results and Discussion

### 2.1 PU Surface Modification

#### 2.1.1 Plasma Treatment Time and PDA Concentration

The wettability of bio‐polymer surfaces plays a critical role in cell adhesion and behavior by regulating protein adsorption, and is cell‐type dependent.^[^ ^13^ ^]^ As fibroblasts are the key drivers of wound healing and regeneration, understanding the impact of increasing hydrophilicity on the fibroblastic response is paramount for both the short and long‐term success of implants.^[^ ^14^ ^]^

To determine the impact of plasma treatment time and/or PDA concentration, and the optimal order of the two treatments on the surface energy of E2A substrates, changes in water contact angle were measured after plasma treatment periods between 15 s and 5 min, and PDA concentrations between 0.005 and 0.5 w v^−1^%. Human foreskin fibroblasts (HFFs) were seeded on modified substrates, and their ability to reduce MTS tetrazolium compound after 48 h of direct contact was assessed as a measure of cell viability.

**Figure 1:**
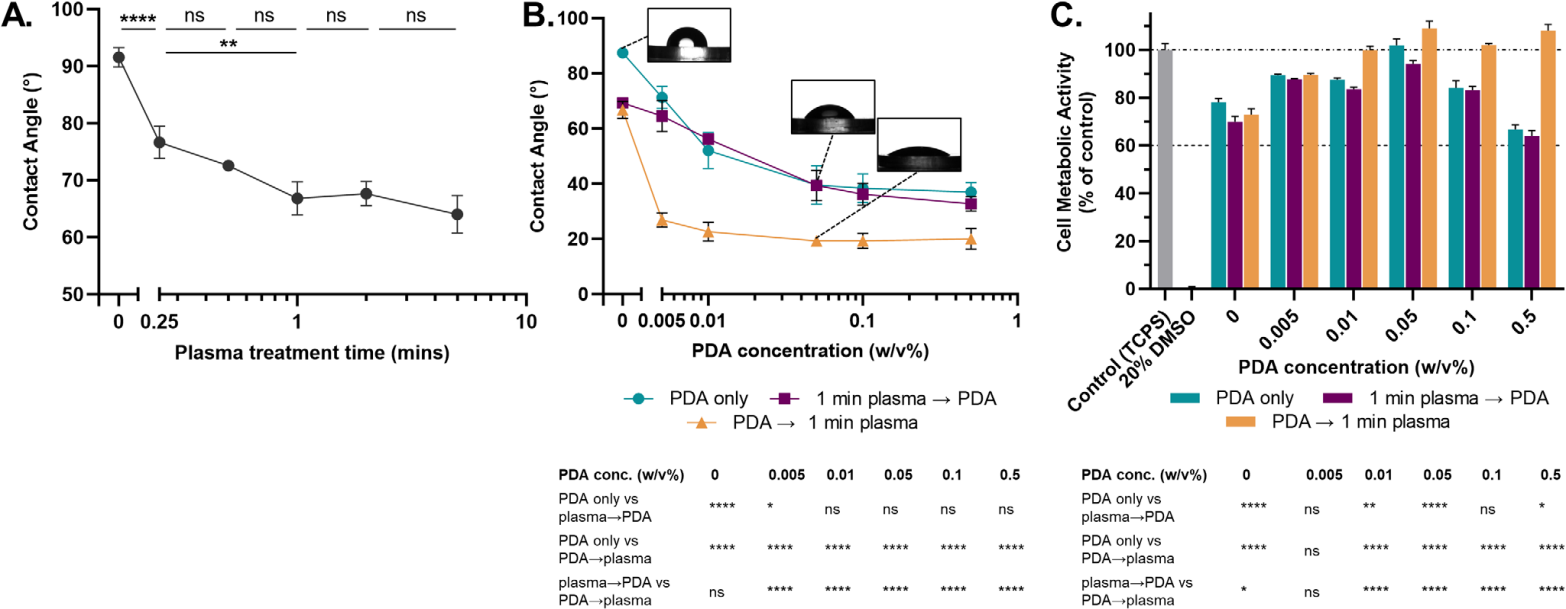
Optimization of silicone‐polyurethane co‐polymer surface modification parameters on surface wettability and cell viability. A) Water contact angle as a function of oxygen plasma treatment time. B) PDA concentration versus contact angle preceding and succeeding 1‐min surface plasma treatment, or on unmodified substrates. C) HFF cell metabolic activity for plasma and PDA‐modified substrates. Graphs: bars or symbols represent the mean and errors bars are ±1SD (*n* = 3). A Tukey's multiple comparisons test was used to compare each mean at each PDA concentration (ns: *p* > 0.05, ^*^ *p* ≤ 0.05, ^**^ *p* ≤ 0.01, ^***^ *p* ≤ 0.001, ^****^ *p* ≤ 0.0001).

The contact angle fell from 92° in the untreated to 67° after 1 minute of oxygen plasma exposure, with no additional benefit beyond 1 min exposure (**Figure** **1**A). Increasing the PDA concentration also resulted in a continuous decrease in contact angle, plateauing at concentrations above 0.05 w v^−1^% (Figure 1B).

In contrast to previous studies on a variety of alternative polymeric materials, it was observed that plasma pre‐treatment may not increase PDA adsorption to the E2A surface regardless of PDA concentration used, evidenced by comparable contact angle and cell viability (Figure 1C) on PDA‐only and plasma pre‐treated PDA substrates.^[^ ^12^, ^15^, ^16^ ^]^ Tukey's multiple comparisons test reveals *p*‐values greater than 0.1 when testing PDA only versus 1‐min plasma before PDA deposition contact angles, for PDA concentrations above 0.005 w v‐1%. However, plasma treatment after PDA coating further modified the surface energy, with the greatest decrease ≈90–19° after 0.05 w v^−1^% PDA deposition. Unmodified E2A displayed a limited cell viability of ≈75% compared to the tissue culture polystyrene (TCPS) control (Figure 1C). Cell viability increased to above 100% with 0.05 w v^−1^% PDA, likely owing to decreased contact angle and increased cell adhesion as described in the following section 2.3. Additionally, sequential PDA and plasma treatment may be protective against unstable cell adhesion at increasing PDA concentrations as previously reported elsewhere, as suggested by the > 100% cell viability in the PDA‐plasma samples when compared to all other datasets above 0.05 w v‐1% PDA (Figure 1C).^[^ ^8^ ^]^ These findings further support that plasma treatment applied after PDA deposition leads to more substantial modifications in surface properties and thereby, the cell response, than the reverse treatment sequence, likely due to direct interaction with the PDA layer. The associated chemical and physical changes are discussed in detail in Section 2.2.

Based on these results, the surface modification conditions chosen to further characterize this study were E2A untreated, E2A with 1‐min plasma treatment, E2A with 0.05 w v^−1^% PDA, and E2A with 0.05 w v^−1^% PDA followed by 1‐min plasma treatment. These conditions allow for the evaluation of the impact – including surface chemistry and roughness, cell adhesion, and ECM production – of plasma and PDA independently and combined, at concentrations optimized for cell viability.

### 2.2 Surface Characterization

#### 2.2.1 X‐Ray Photoelectron Spectroscopy (XPS)

XPS was used to determine the composition of the material surfaces down to 10 nm. XPS of E2A surfaces was performed 6 hours after modification to allow sufficient time for vacuum drying. Both the XPS survey and high‐resolution carbon region scan were used to detect the composition of material surfaces and relative abundances of these components. Stoichiometric amounts of elements in the pure elastomer are not available for comparison owing to the patented technology.

**Figure 2:**
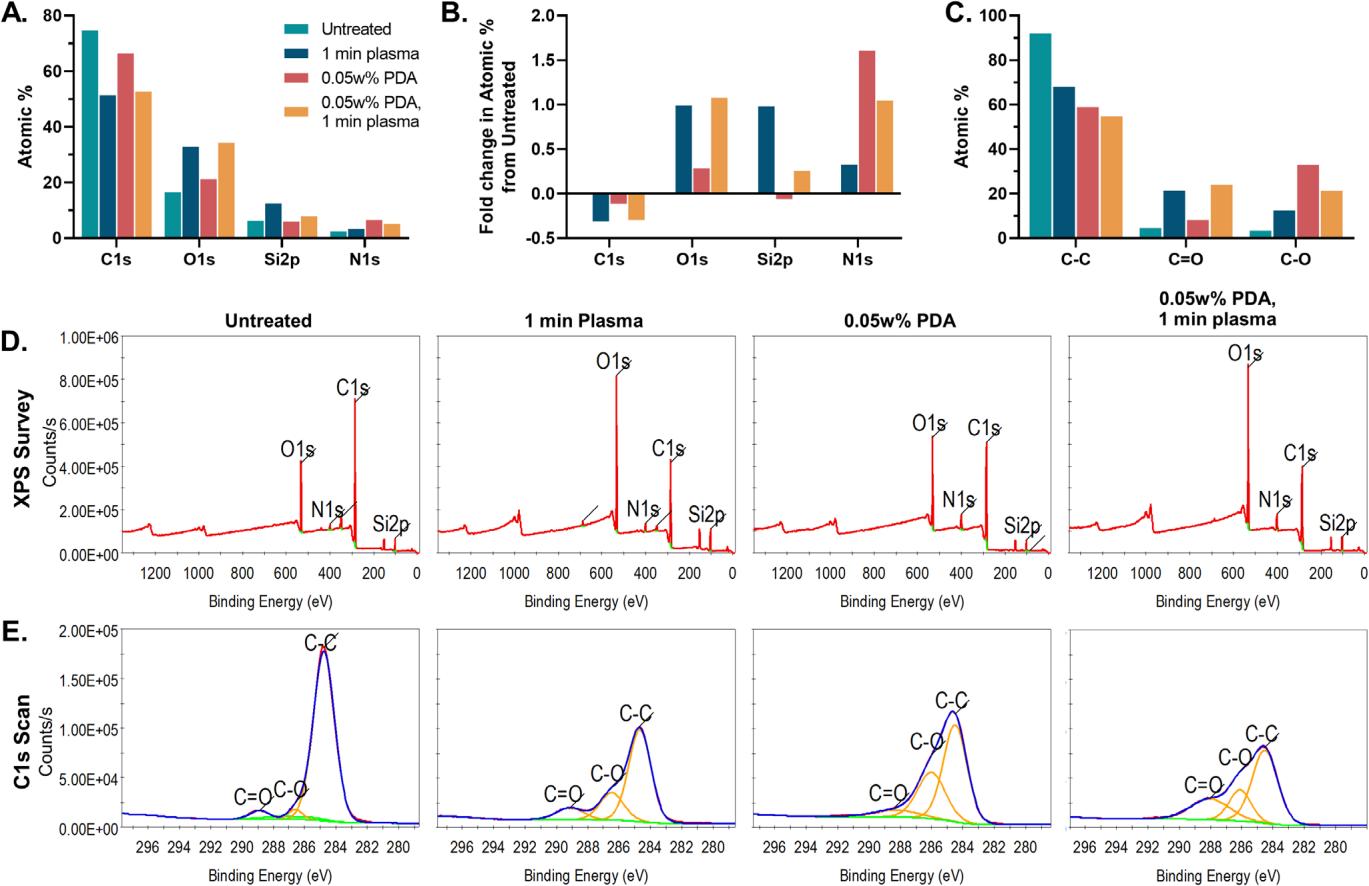
X‐ray photoelectron spectroscopy (XPS) surface analysis of modified polyurethane co‐polymers. A) Atomic percent of species present, and B) the change fold from untreated substrates. C) The atomic percent breakdown of the carbon region (C 1s) region. D) XPS survey, and E) C 1s scan for each substrate.

Samples that underwent oxygen plasma treatment showed an increase in overall oxygen percentage, with a 0.99–1.08‐fold increase in atomic percent compared to untreated (**Figure** **2**A,B). C═O bonds in these samples were also at least 3.6 times greater after plasma treatment. A significantly greater amount of nitrogen was detected on PDA‐modified samples, due to the additional nitrogen deposited on the surface after polydopamine coating. The C─O bonds were also higher in PDA samples, with the lowest number of detectable C─C bonds of any condition (Figure 2C). XPS analysis verified there is a substantial difference in surface chemistry and available functional groups between the different surface treatments, with potential to thereby impact cell adhesion and function. These findings are consistent with previous reports showing that variations in polydopamine surface chemistry, particularly in oxygen‐ and nitrogen‐containing functional groups, can influence cell adhesion and function through modulation of protein adsorption and integrin engagement.^[^ ^17^, ^18^ ^]^

#### 2.2.2 PDA and Plasma‐Induced Surface Roughness

**Figure 3:**
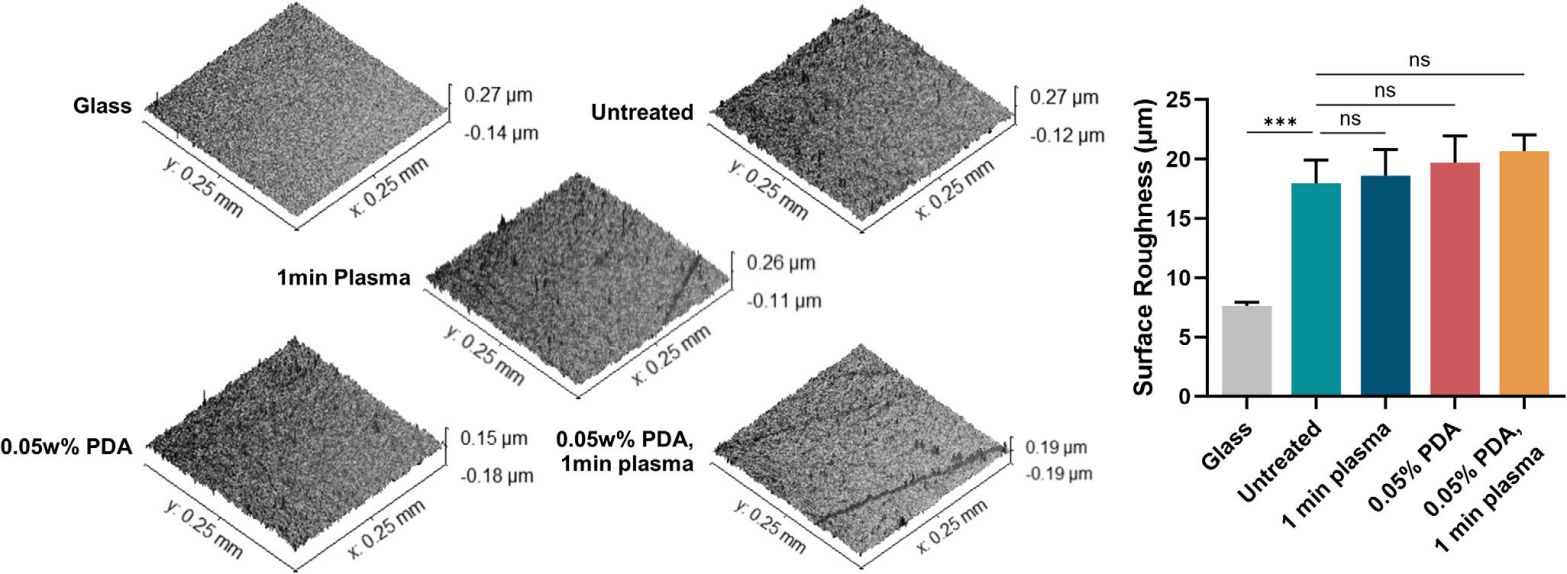
Surface roughness of the unmodified and modified E2A substrates and glass coverslips used in this study. Surface roughness is presented as Sa; the arithmetical mean height of an area. Errors bars are ±1SD (*n* = 3).

To detect microstructural changes induced by plasma oxidation, optical profilometry was conducted on each sample within 1 hour of plasma treatment. No significant change in surface roughness was found between E2A samples and the untreated control (**Figure** **3**), indicating the increase in hydrophilicity is chemically‐driven rather than a result of altered surface morphology from either polydopamine deposition or plasma treatment.

#### 2.2.3 Surface Stability

**Figure 4:**
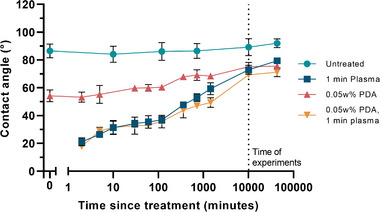
Change in contact angle (hydrophobic recovery) after PDA deposition and/or oxygen plasma treatment over time. The *x*‐axis represents minutes from plasma treatment or drying after PDA deposition, and the error bars are ±1 SD (*n* = 3).

To assess the lifetime of the surface treatments and better understand the surface energy trends over time, the contact angle was measured at time points beginning immediately after treatment and continuing up to one month post‐treatment. The largest impact on surface energy occurred immediately after PDA deposition and/or plasma oxidation. The contact angle of plasma‐modified surfaces trends toward the unmodified surfaces over the month due to hydrophobic recovery (**Figure** **4**), however, the hydrophilicity of treated samples are expected to fare better when stored in an air‐tight and sterile environment after modification to avoid transfer of airborne contaminants, or at a lower temperature to decrease polymer chain mobility.^[^ ^19^ ^]^ Using the treated biomaterial shortly after plasma oxidation is therefore crucial to maximize the impact of increased hydrophilicity, as the initial surface energy is most conducive to enhancing cell adhesion and functionality. However, despite the contact angle only being 16% lower than untreated E2A at 7 days – the time of experiments, and a feasible timeframe for implant processing and sterilization – differences in cell adhesion and function were observed, indicating a lasting impact on cell behavior that may arise from the compounding effects of increased surface wettability and differences in surface chemistry.

### 2.3 Fibroblast Attachment to Modified Surfaces

#### 2.3.1 Fibroblast Adhesion

Cell adhesion is one of the crucial steps in the wound healing process, allowing wound closure through remodeling and angiogenesis. To evaluate cell adhesion on the treated and control surfaces, HFFs were seeded at a low density (6000 cells mm^−2^), as higher seeding densities resulted in cell migration and clumping, leading to uneven distributions and inaccurate cell density readings. 24 hours after cell seeding, samples were rinsed, fixed and fluorescently labeled; cell density and Feret's diameter (longest length of each individual cell) was measured to score and characterize cell attachment across the surfaces.

**Figure 5:**
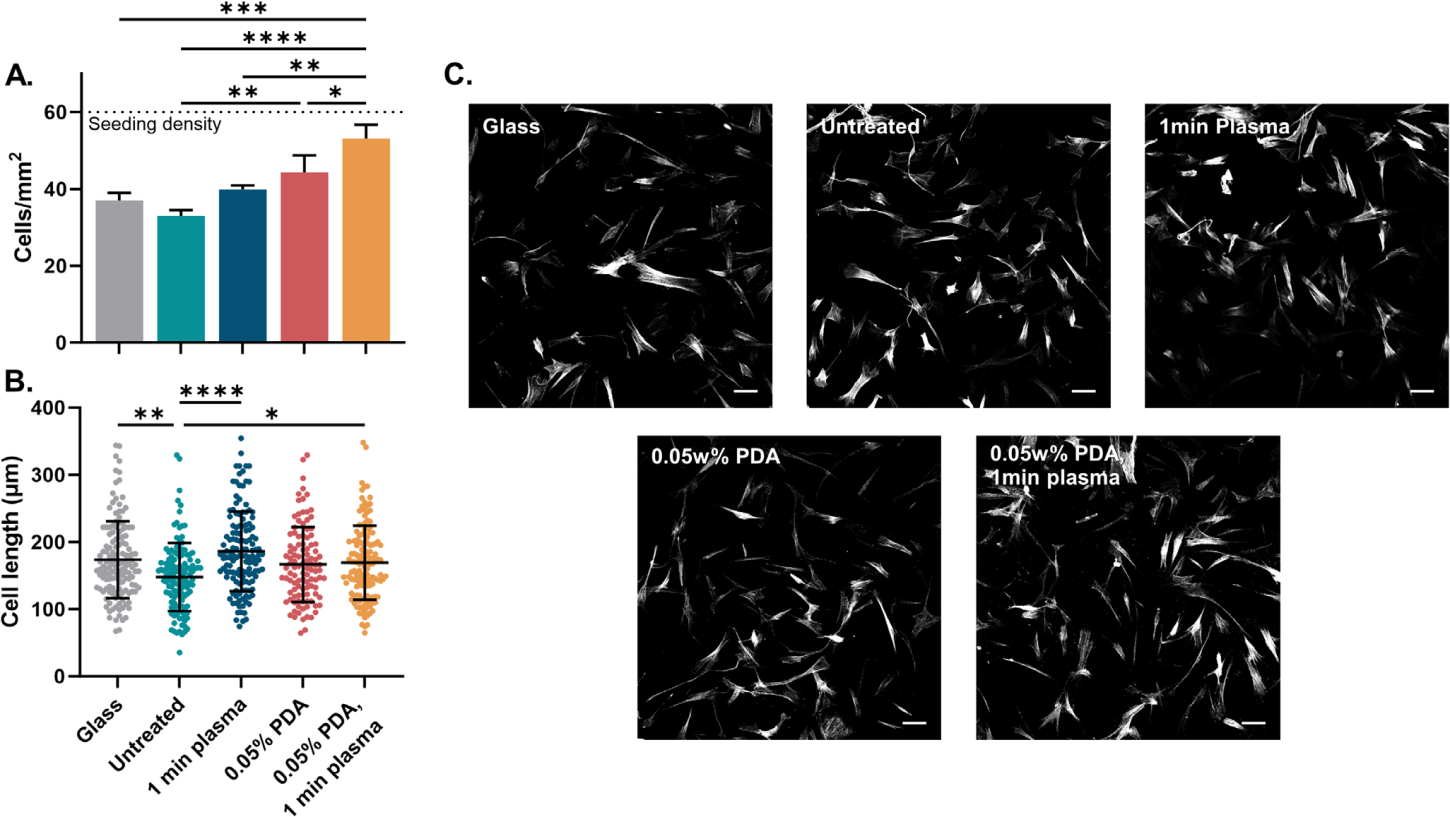
Fibroblast adhesion assay on glass coverslip control, and unmodified and modified E2A substrates. A) Average adhered cell density after 24‐h incubation (*n* = 3), B) Feret's diameter, Plot graphs display individual cell length measurements (*n* = 135). Centre bars indicate the mean length. Error bars are ±1 SD (^*^ *p*≤0.05; ^**^ *p*≤0.01 ^***^ *p*≤0.001; ^****^ *p*≤0.0001). C) Actin staining of adhered fibroblasts at 24 h after low density seeding. Scale bar = 100 µm.

A significant difference in the number of adhered cells was observed across the surfaces with the lowest on untreated E2A (33.0 cells mm^−2^) and the highest on the PDA and plasma‐treated E2A (53.2 cells mm^−2^, *p*<0.0001), ≈89% of the seeded cell population (**Figure** **5**A). HFFs have an elongated spindle morphology and the Feret's diameter of the cells was reduced on the unmodified substrate compared to the glass coverslip, but increased 22% on the PDA and plasma‐modified E2A (142–173 µm, p = 0.0191), compared to the unmodified E2A (Figure 5B). Interestingly, the plasma‐only treated sample also showed a significant increase in cell length, but no change in cell adhesion density, suggesting that additional oxygen‐containing functional groups introduced by plasma treatment may promote cell spreading through enhanced surface hydrophilicity and cytoskeletal reorganization. An increase in both the number and length of the cells indicates enhanced adhesion of the fibroblasts in the first 24 h of exposure.

#### 2.3.2 Extracellular Matrix Production

**Figure 6:**
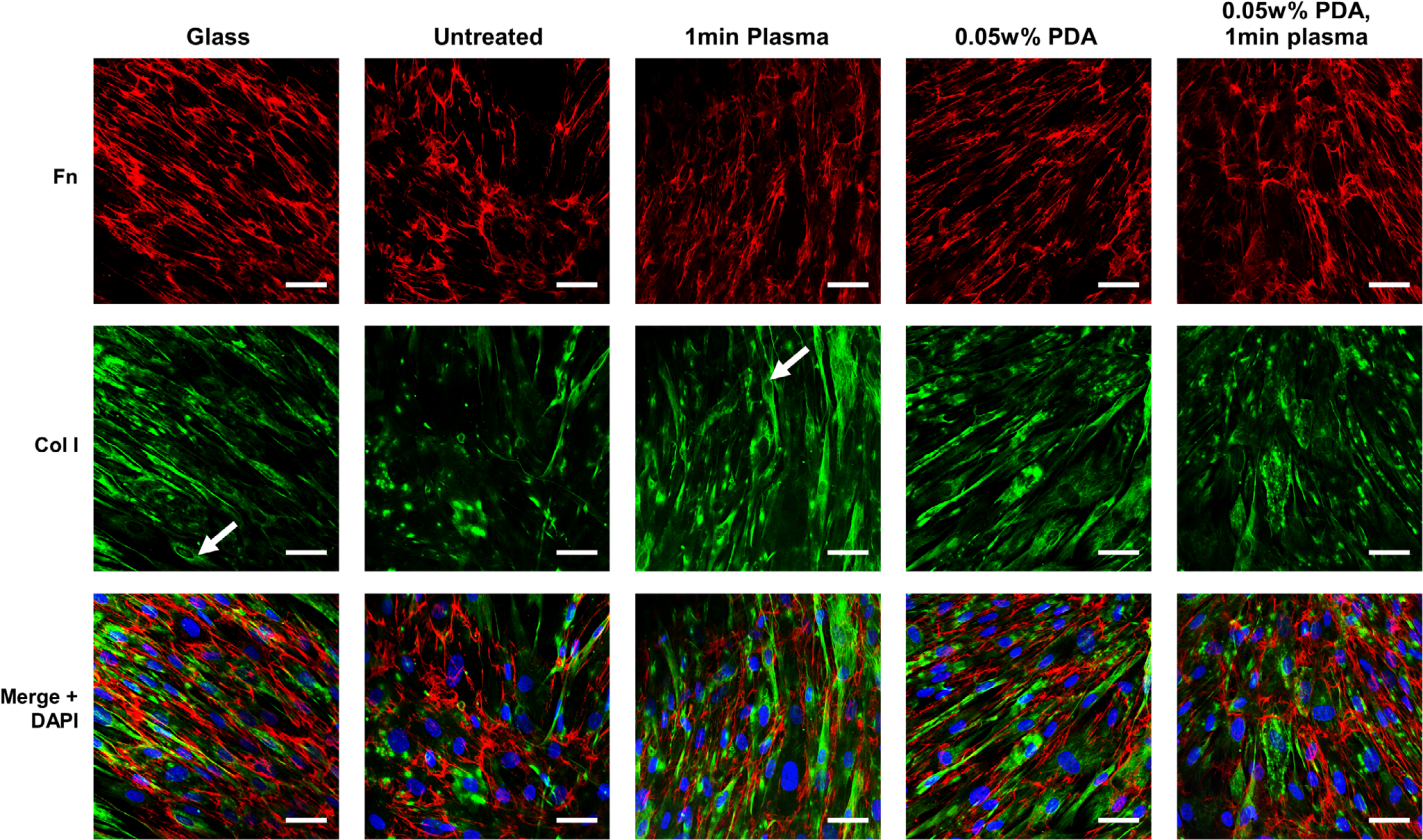
Cellularized ECM at day 7, red: fibronectin, green: collagen I, and blue: DAPI. Arrows indicate the perinuclear distribution of collagen I. (scale bar = 50 µm).

Extracellular matrix (ECM) components form a tissue‐specific natural polymer network that supports tissue architecture and regulates cell adhesion and migration.^[^ ^20^ ^]^ Recruited fibroblasts are responsible for the deposition of the fibronectin‐rich provisional matrix which templates the more permanent ECM comprising collagens.^[^ ^14^ ^]^ To provide an understanding of the contribution of the surface modifications on fibroblast ECM activity, the amount of intra‐ and extra‐cellular collagen I and fibronectin was measured (**Figure** **6**).^[^ ^14^ ^]^ Cells were incubated on the various surfaces for 7 days and formed a monolayer, followed by fixation and immunostaining with fibronectin and collagen I antibodies, as well as DAPI.

As displayed in Figure 6, HFFs produced ECM on all substrates. At 7 days, the majority of collagen I appears to be intracellular, with a perinuclear distribution around the nucleus (Figure 6, see arrows). From the fluorescent images, an increase in cell alignment – defined as similar orientation of the cell's long axis – was observed in treated samples compared to untreated E2A controls. Untreated surfaces show a more irregular orientation and decreased collagen I intensity.

**Figure 7:**
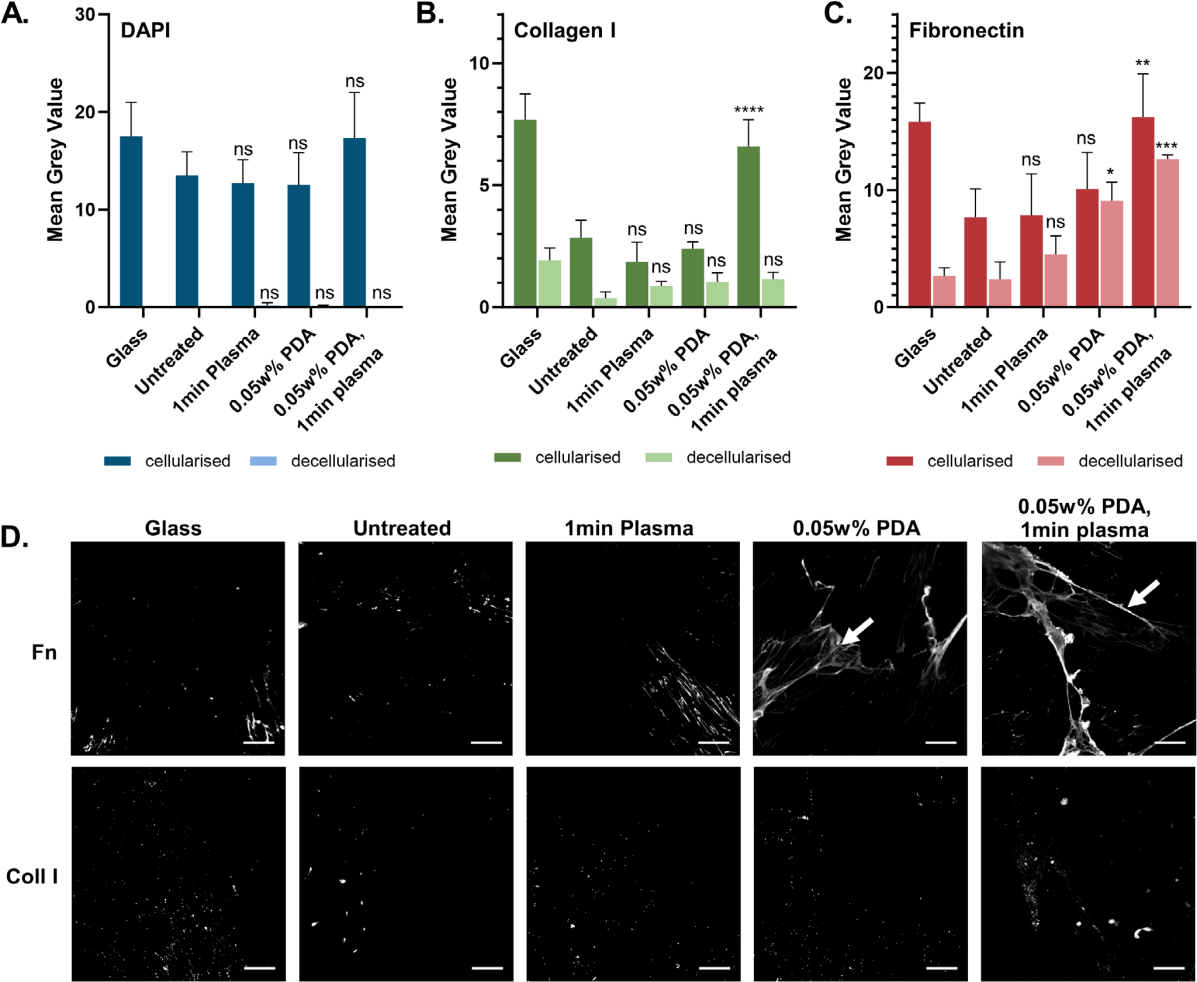
Mean gray value of cellularised and decellularised ECM after 7‐day incubation A) DAPI, B) Collagen I, C) Fibronectin (*n* = 3). Error bars are ±1 SD, statistical difference indicated for each E2A treatment condition compared to the untreated E2A samples, cellularised and decellularised respectively (ns: *p* > 0.05, ^*^ *p* ≤ 0.05, ^**^ *p*≤0.01, ^***^ *p* ≤ 0.001, ^****^ *p* ≤0.0001). D) decellularized IF images. Arrows indicate fibronectin fibrils observed on the PDA‐treated surfaces (scale bar 10um).

To evaluate the secreted extracellular ECM proteins, cells cultured on various surfaces were treated with ammonium hydroxide to remove soluble cell components whilst preserving the secreted and deposited ECM proteins. Fluorescence intensity was measured through the mean gray value; the sum of the gray values of all the pixels divided by the number of pixels, for both cellularized and decellularized ECM. The success of the cell‐solubilization technique was evidenced by decreased DAPI fluorescence intensity (mean gray value of ≈0) after treatment (**Figure** **7**A). Plasma treatment after PDA deposition produced an increase in cellularised fibronectin (p = 0.0271) and collagen I (*p*<0.0001) compared to the PDA‐only substrates (Figure 7B,C). Long fibronectin fibrils were observed on the PDA‐treated surfaces (Figure 7D, see arrows). The level of extracellular fibronectin was increased compared to the predominantly intracellular collagen I, although a longer cell culture incubation period may alter the ratios and allow greater deposition of polymerized ECM proteins. However, at 7 days it is evident that plasma and PDA modifications allow more rapid ECM production, and thereby a faster wound healing response.

Ultimately, both PDA deposition and plasma treatment can modify the fibroblast response to polyurethane co‐polymers for greater cell adhesion and ECM production. In addition to the fibroblast response, the hemocompatibility of cardiac medical devices is an essential requirement for optimal function. Despite their mechanical compatibility and material stability, the hydrophobicity of untreated PUs leads to significant biofouling, which may result in thrombosis and ultimately implant failure or patient death.^[^ ^21^ ^]^ Therefore, higher energy surfaces may improve blood compatibility through both a short‐term reduction in biofouling and long‐term endothelialization, creating a physical barrier between the device surface and the blood.

Although this study only assessed the short‐term impact on one cell line and further research into the effects on complex tissue structures over longer periods and hemocompatibility is required, PDA and plasma surface engineering provide a promising, versatile approach for better tissue integration of flexible biomaterials.

### 2.4 Whole Blood Adhesion to Modified Surfaces

Surface modification of hydrophobic PU co‐polymers is critical to enhancing endothelial cell attachment required for the long‐term success of blood‐contacting devices. However, this may reduce the material's natural capacity to prevent protein adsorption and platelet adhesion. To determine the impact of PDA and plasma modifications on haemocompatibility, citrated whole blood from three different healthy donors was incubated on samples for 40 min at 30 RPM on an orbital shaker, and then washed, fixed, and stained with phalloidin to identify F‐actin for analysis. Red blood cells (RBCs) were detected via glutaraldehyde‐induced autofluorescence in the 575 nm emission range.^[^ ^22^, ^23^ ^]^ Similar to the fibroblast studies, uncoated glass coverslips were used as a control, where very low numbers of platelets have been demonstrated to adhere in the absence of bioactive coatings.^[^ ^24^ ^]^ One donor's blood was also added to biopsy‐punched samples of Tygon E‐3603; a phthalate‐free thermoplastic elastomer tubing very similar to that used in extracorporeal membrane oxygenation (ECMO) circuits as an additional negative control.

**Figure 8:**
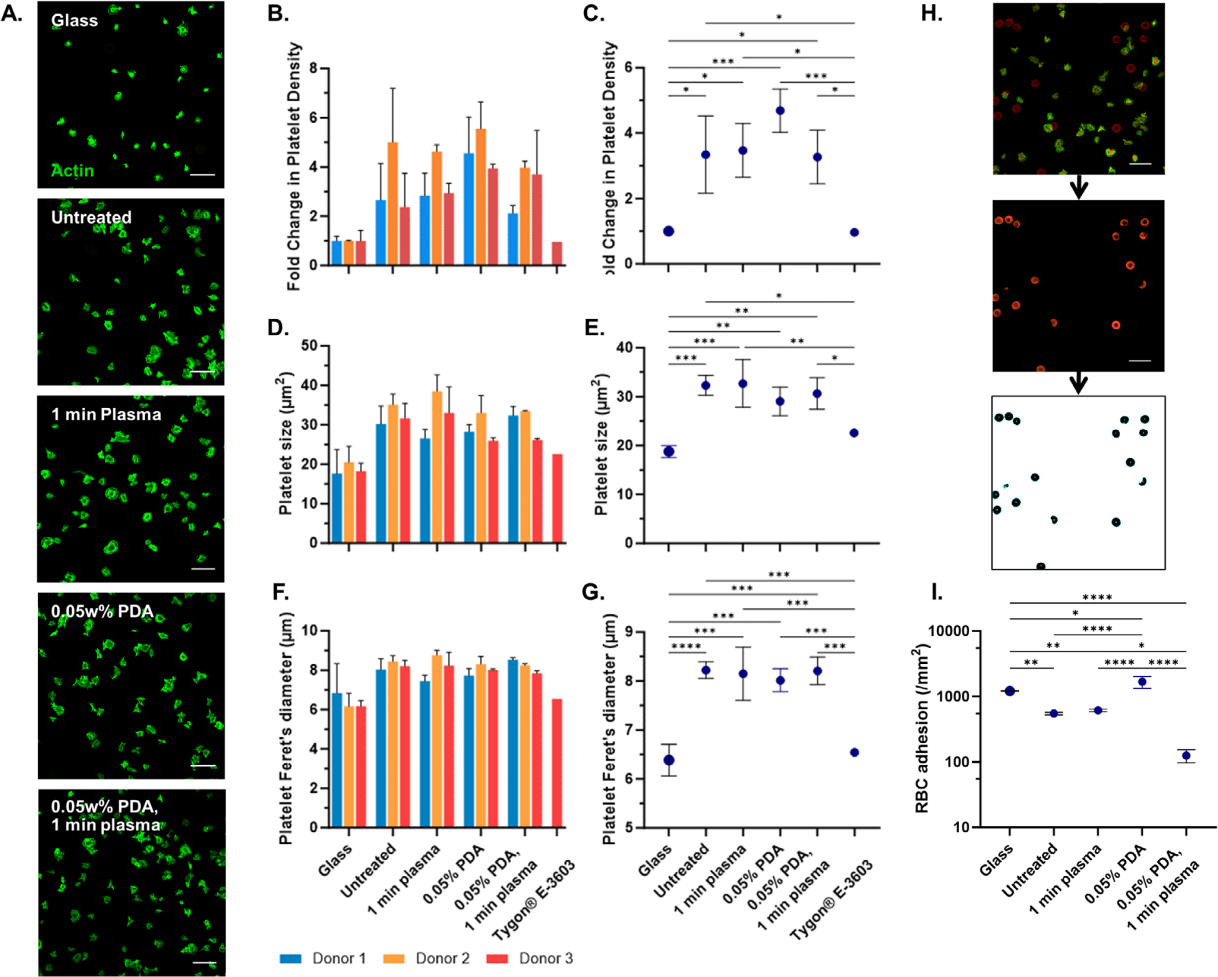
A) Whole blood platelet adhesion, stained for F‐actin (scale bar 10 µm) B,C) donor‐specific and combined average fold change in adhered platelet number, D,E) donor‐specific and combined average adhered platelet size, F,G) donor‐specific and combined average adhered platelet diameter, H) method of fluorescence subtraction from background autofluorescence used to detect red blood cell density (scale bar 10 µm), and I) combined average red blood cell adhesion density. Three donors were used across three technical replicates per donor. Error bars are ±1 SD (^*^ *p* ≤ 0.05; ^**^ *p* ≤ 0.01 ^***^ *p* ≤ 0.001; ^****^ *p* ≤ 0.0001).

Platelets were detected on all surfaces (**Figure** **8**A). To account for the range in natural platelet count in whole blood, results were standardized by the number of platelets adhered to the glass coverslip control and ECMO tubing. Untreated E2A displayed a 3‐fold increase in platelet density compared to the low‐adherent glass coverslip and was consistent across the treated E2A surfaces (*p* > 0.05) (Figure 8B,C). Neither platelet volume nor Feret's diameter was different across the E2A surfaces, but was significantly increased compared to the glass/ECMO control (Figure 8D–G). Specific marker of platelet activation may provide further insight into activation status across the material surfaces, however platelet size has been used previously as a measure of activation state, and in this instance, also shows no statistical difference between E2A experimental treatments.^[^ ^25^ ^]^ Interestingly, the number of adhered red blood cells was significantly reduced when PDA and plasma were combined, likely as a result of the introduction of additional functional groups, though this observation should be interpreted cautiously given the limited sample size. In summary, these findings suggest that PDA and plasma treatments may not only enhance hemocompatibility through more rapid wound healing, their effects on platelet adhesion before complete endothelialization are likely minimal, leading to an overall more haemocompatible biomaterial.

Future studies should investigate the mechanisms underlying the sequence‐dependent effects of PDA and plasma treatment, with a particular focus on molecular‐level interactions between surface chemistry, protein adsorption, and signaling pathways. Additionally, extending the evaluation to include diverse cell types and longer‐term in vitro or in vivo models would provide a more comprehensive assessment of the treated material's regenerative potential.

## 3 Conclusion

In summary, this study identifies an enhanced combination of PDA and plasma modification on an established flexible biomaterial to counteract the natural hydrophobicity and enhance critical tissue integration; for Elast‐Eon E2A, this is 0.05 w v‐1% PDA followed by 1‐min plasma treatment. A significant increase in fibroblast adhesion and ECM production was observed compared to untreated controls, likely due to the changing surface chemistry and wettability, without a significant increase in platelet adhesion or size. The comprehensive material and surface characterization, and in vitro cell culture results presented here provide a better understanding of surface engineering options for elastomeric biomaterials and contribute to enhanced medical implants for better patient outcomes.

## 4 Experimental Section

### E2A Solvent Casting

E2A pellets were dissolved at 8 w v^−1^% in *N,N*‐dimethylacetamide (DMAc) (Sigma–Aldrich, 38840–1L‐F) at 70 °C overnight in a fumed oven. The solution was stirred every hour the following day until a homogenous solution was obtained, then a ≈10 mm thick layer was poured into a pre‐warmed glass petri dish. Upon complete evaporation ≈3 days later, a ≈1 mm film of Elast‐Eon remained, which was cut into 8 mm round discs using a biopsy punch.

### Polydopamine Deposition

UltraPure Tris Buffer (Astral Scientific, BIO3094T‐1KG) was made up to a 10 mm solution and titrated with hydrochloric acid to a final pH of 8.5. Dopamine hydrochloride (Sigma–Aldrich, H8502) was dissolved in Tris buffer to the desired concentration and added to the substrates for 24 h at room temperature. The samples were then washed three times with deionized water, with 5 min on a plate shaker (80 RPM) between each rinse.

### Plasma Oxidation

Samples were dried for at least 1 h at 40 °C before plasma treatment. Plasma treatment occurred under vacuum using the Harrick Plasma High Power Expanded Plasma Cleaner and oxygen gas at 450–500 mTorr at high radio frequency (RF) level.

### Sample Sterilization

Samples were sterilized under UV light for 10 min each side, and transferred to sterile 48‐well tissue culture plates immediately prior to cell culture experiments.

### Contact Angle

Water contact angle was measured using the OCA15 Pro Video‐Based Optical Contact Angle Measuring System (DataPhysics); a 10 µL deionized water droplet was syringed at 2 µL s^−1^ onto the substrate surface, and the contact angle measured after 10 s using elliptical fitting on the SCA20 software (DataPhysics, v. 3.50.1). For treatment lifetime analysis, plasma treated samples were measured instantly, allowing a 2‐min measure of contact angle. Three samples per condition were measured, per timepoint, from untreated (0 s) to 1 month.

### X‐Ray Photoelectron Spectroscopy (XPS)

X‐ray photoelectron spectroscopy (XPS) was performed on a Thermo Scientific Nexsa Surface Analysis System equipped with a hemispherical analyzer. The incident radiation was monochromatic Al Kα X‐rays (1486.6 eV) at 72 W (6 mA and 12 kV, 400 × 250 µm^2^ spot). Survey (wide) and high‐resolution (narrow) scans were recorded at analyzer pass energies of 150 and 50 eV and step sizes of 1.0 and 0.1 eV, with 5 and 10 scans, respectively. The base pressure in the analysis chamber was less than 5.0 × 10^−9^ mbar. A low‐energy dual‐beam (ion and electron) flood gun was used to compensate for surface charging. Data processing was carried out using Avantage software (v5.9931) and the energy calibration was referenced to the main line of C 1s at 284.8 eV. The background used for curve fitting was Smart. Chemical species were identified using the inbuilt XPS Knowledge View.

### Optical Profilometry

Optical profilometry was performed using the Bruker X‐200 optical profilometer with a scan surface area of 763 µm by 636 µm and Z range between 3–7 µm to cover all amplitudes. The data was analyzed using Gwyddion v2.66 software. Data was leveled using the remove polynomial background function, and the mean roughness (Sa) was determined for the entire region of three samples per condition. A 3D topography was generated for the central 250 µm by 250 µm region.

### Cell Seeding and Culture

Human foreskin fibroblasts (HFFs) (ATCC HFF‐1, In Vitro Technologies, ATCSCRC1041) were cultured in Dulbecco's Modified Eagle's Medium DMEM with 4.5 g L^−1^ glucose supplemented with 15% fetal bovine serum (FBS), split 1:3‐1:5 twice weekly, and used between passages 8–12. Prior to seeding, cells were washed three times with phosphate buffer solution (PBS), detached with Tryple express, centrifuged at 300 ×g for 5 min, re‐suspended in fresh culture medium, and manually counted using a 1:1 ratio of trypan blue and a hemocytometer. Unless otherwise specified, 8000 cells cm^−2^ were seeded on each substrate within the 48‐well plate containing 300–350 µL of growth medium and incubated at 37 °C in a 5% CO_2_, humidified incubator.

### Cell Viability

To determine the viability of the fibroblasts on the substrates, a direct‐contact MTS assay was performed with cell culture media and 20% Dimethylsulfoxide (DMSO) in cell culture media as the negative and positive control, respectively. After 48 h incubation, the original media was replaced with 300 µL fresh serum‐free media containing 10% MTS reagent (Abcam, ab197010). An additional 300 µL serum‐free media without MTS was added to empty wells to account for media colour during analysis. The plate was incubated for 2 h in the dark and shaken briefly before the media from each well was transferred in 100 µL aliquots to a new 96‐well plate, and absorbance at OD = 490 nm captured by the Victor Nivo Multimode Plate Reader (PerkinElmer).

### Cell Adhesion

HFFs were seeded at 6000 cells cm^−2^ for 24 h, after which the growth medium was removed, the cells were gently rinsed with PBS three times, fixed in 4% paraformaldehyde (PFA) in PBS for 20 min, and permeabilized by 0.1% Triton‐X in PBS for 90 s, both followed by three PBS washes. The fixed samples were blocked in 10% FBS in PBS at room temperature for 45 min or at 4 °C overnight. Samples were stained with ActinGreen 488 ReadyProbes (Invitrogen, R37110; 2 drops mL^−1^) for 1 h in the dark, rinsed, and mounted on glass slides with Prolong Diamond Mounting Solution with DAPI (Thermo Fisher Scientific, P36971).

### Extracellular Matrix Production

To assess fibroblast ECM production and secretion across the substrates, cells were labeled for collagen I and fibronectin. After a 7‐day incubation, fixed and blocked samples were incubated with anti‐collagen I polyclonal primary antibody from rabbit (Sigma–Aldrich, SAB4500362; 5 µg mL^−1^) and anti‐fibronectin monoclonal primary antibody from mouse (Sigma–Aldrich, F7387; 5 µL mL^−1^) for 1 h at room temperature. The cells were carefully washed three times with PBS and incubated with secondary antibody Alexa Fluor 488 Goat anti‐Rabbit (Thermo Fisher Scientific, A32731; 2 µg mL^−1^) and Alexa Fluor 555 Rabbit anti‐mouse (Thermo Fisher Scientific, A32727; 2 µg mL^−1^) for 45 min at room temperature in the dark. The samples were washed three times with PBS and mounted on glass slides with Prolong Diamond Mounting Solution with DAPI (Invitrogen, P36966).

To determine the intra‐ versus extra‐cellular concentration of fibronectin and collagen I, cells were removed with ammonium hydroxide solution following methods described by Hellewell, et al.^[^ ^26^ ^]^ After the culture media was removed and three PBS rinses, cells were covered with 20 mm ammonium hydroxide in de‐ionized H_2_O and incubated at room temperature for 5 min, with gentle agitation every minute. The remaining insoluble ECM layer was washed in large amounts of de‐ionized H_2_O at least four times prior to fixation and staining as above.

### Whole Blood Adhesion

Fresh human whole blood was obtained from healthy donors with informed consent, in accordance with the guidelines of Monash University Human Ethics Committee (Project ID: 35867). Donors had not taken anticoagulant, anti‐inflammatory or antibiotic medication 72 h prior to donation. Blood was drawn via standard venepuncture into polypropylene syringes at 3.2% w v^−1^ trisodium citrate and stored at 37 °C until use. Blood was used within 4 h of collection.

Whole blood was added directly on top of each sample in a 48‐well plate and incubated at 37C on a dynamic shaker at 30 RPM for 40 min. Blood was removed and samples were gently washed with PBS three times before fixation with 2.4% glutaraldehyde and 0.24% PFA in PBS for 2 h at room temperature. After each well was rinsed three further times, it was filled with PBS and kept at 4 °C until further use.

Fixed whole blood was labeled with ActinGreen 488 ReadyProbes (Invitrogen, R37110; 2 drops mL^−1^) and Alexa Fluor anti‐human CD41/CD61 antibody from stock (362806, Clone: PAC‐1, 10 µL mL^−1^) for 1 h in the dark. No signal was detected when stained with Anti‐Fibrin Antibody (Sigma, MABS2155; 10 µL mL^−1^) with secondary antibody Alexa Fluor 555 Rabbit anti‐mouse as previously described.

### Confocal Laser Scanning Microscopy

The fluorescently labeled substrates were imaged with an Olympus FLUOVIEW FV3000 confocal laser scanning microscope equipped with a digital camera. Three‐to‐five images were taken per substrates at 20x objective for HFFs and 60x objective for whole blood using identical acquisition settings across all samples. The number of cell nuclei, and the fluorescence intensity of the proteins was measured automatically using Fiji ImageJ.^[^ ^27^ ^]^ Due to cell overlap, ferrets diameter was measured manually by adding a 4 × 4 grid to each 1:1 image, and measuring the longest length of the cell at each of the nine nodes. Values were presented as the mean of each experimental repeat, with error bars representing the standard deviation between all three repeats.

### Statistical Analysis

Ordinary one‐way or two‐way unpaired ANOVA test followed by Tukey's multiple comparisons test was performed using GraphPad Prism 9 (v. 9.4.1, GraphPad Software). Statistical significance was considered achieved when *p* < 0.05. (ns: *p* > 0.05, ^*^ *p*≤0.05, ^**^ *p* ≤ 0.01, ^***^ *p*≤0.001, ^****^ *p*≤0.0001). Data was presented as mean ± 1 SD.

## Conflict of Interest

The authors declare no conflict of interest.

## Data Availability

The data that support the findings of this study are available from the corresponding author upon reasonable request.
